# Supplementary material for: Advancing mRNA subcellular localization prediction with graph neural network and RNA structure
Source: Bioinformatics. 2024 Aug 12;40(8):btae504. doi: 10.1093/bioinformatics/btae504 (PMC11361792; doi:10.1093/bioinformatics/btae504)
Supplement: btae504_Supplementary_Data [file btae504_supplementary_data.pdf]

Advancing mRNA subcellular localization prediction with graph neural network and RNA structure

1 Supplementary Tables

Table S1: The encoding schemes of NCP (nucleotide chemical property), EIIP (electronion interaction pseudopotentials) and ANF (accumulated nucleotide frequency).

	Nucleotide types	NCP	EIIP	ANF
A	[0,1,0,0,0]	[1,1,1]	0.1260	d
G	[0,0,1,0,0]	[1,0,0]	0.0806	d
C	[0,0,0,1,0]	[0,1,0]	0.1340	d
T/U	[0,0,0,0,1]	[0,0,1]	0.1335	d

Table S2: Allocator's parameter setting.

Parameter	Setting
Number of GIN input features	10
Number of MLPs input features	96/1364
Hidden layer dimension	64
Number of output classes	6
Dropout rate	0.1
Activate function	Relu
Optimization algorithm	Adam (learning rate:0.0003)
Batch size	128
Batch normalization	True

Table S3: Performance for each label/localization between DM3Loc and allocator.

Localization	MCC		Accuracy	
	DM3Loc	Allocator	DM3Loc	Allocator
Nucleus	0.3483	0.3309	0.6817	0.7362
Exosome	0.0422	-0.0026	0.7371	0.9880
Cytosol	0.1859	0.3014	0.5783	0.6563
Ribosome	0.3531	0.3054	0.6811	0.7145
Membrane	0.2999	0.2292	0.7805	0.8083
ER	0.0241	0.1393	0.8614	0.8864

Table S4: Training-validation results vs. 5-fold and 10-fold cross-validation results.

Strategy	Acc_{exam}	Average precision	Coverage	One-error	Ranking loss	Hamming loss
Training-validation	0.6473	0.7138	4.4111	0.7560	0.3213	0.2049
5-fold cross-validation	0.6470	0.7039	4.3891	0.7725	0.3170	0.2118
10-fold cross-validation	0.6463	0.7044	4.4056	0.7793	0.3224	0.2129

2 Supplementary Material

2.1 ANF description

The ANF encoding contains the information and distribution of each nucleotide in the RNA sequence, and the calculation formula is as follows:

$$d_i = \frac{1}{|s_i|}\sum_{j=1}^{l} f\left(s_i\right),\ \ f(q) = \begin{cases} 1\ if\ s_i = 1, \\ 0\ other\ case \end{cases} \tag{1}$$

where l represents the length of the mRNA sequence, and s_i is the length of the i-th prefix sequence fragment$\{s_1,\ s_2,\ldots,s_i\ \}$ in the mRNA sequence, $q\ \epsilon\ \{$A,C,G,U$\}$. For instance, when considering the sequence 'UCGGUCAUCG', the ANF encoding values for 'U' at positions 1, 5, and 8 in the sequence are 1 (1/1), 0.4 (2/5), and 0.375 (3/8), respectively.

2.2 Performance evaluation metrics

To evaluate Allocator's overall performance, we utilized six evaluation metrics designed explicitly for multi-label problems, including example-based accuracy (Acc_{exam}), average precision, coverage, one-error, ranking loss, and hamming loss. These metrics offer a comprehensive assessment of Allocator's capabilities across all six labels/locations. The descriptions of these metrics are provided below:

$$Acc_{exam} = \frac{1}{t}\sum_{i=1}^{t} \frac{|P_i \cap Y_i|}{|P_i \cup Y_i|} \tag{2}$$

$$\begin{gathered} Average\ Precision = \frac{1}{t}\sum_{i=1}^{t} \frac{1}{|Y_i|} \\ \sum_{y \in Y_i} \frac{|\{y'\,|Rank_f\left(x_i, y'\right) \leq Rank_f\left(x_i, y\right), y' \in Y_i\,\}|}{Rank_f\left(x_i, y\right)} \end{gathered} \tag{3}$$

$$Coverage = \frac{1}{t}\sum_{i=1}^{t} max_{y' \in Y_i} Rank\left[f\left(x_i, y'\right)\right] - 1 \tag{4}$$

$$One - error = \frac{1}{t}\sum_{i=1}^{t} I\left(\arg max_{y' \in Y_i} f\left(x_i, y'\right) \notin Y_i\right) \tag{5}$$

$$\begin{gathered} Ranking\ Loss = \frac{1}{t}\sum_{i=1}^{t} \\ \frac{1}{|Y_i|\left|\bar{Y}_i\right|} I\left(f\left(x_i, y'\right) \leq f\left(x_i, y''\right), y' \in Y_i,\ y'' \in \bar{Y}_i\right) \end{gathered} \tag{6}$$

$$Hamming\ Loss = \frac{1}{t}\sum_{i=1}^{t} \frac{1}{q} |P_i \Delta Y_i| \tag{7}$$

where $f(\cdot)$ represents the classifier; Y_i and $\overline{Y}_i$ represent the set of real labels and the complement of this set, respectively; P_i represents the set of predicted labels; (x_i, y_i) $\{1 \leq i \leq t\}$ represents a multi-label instance;

$Rank_f(x, y)$ indicates the descending ranking of y in Y; $|\cdot|$ indicates the cardinality of the set, $q = |Y_i|$; Δ is the symmetric difference between two sets; and $I(\cdot)$ indicates the count that satisfies the condition.

3 Supplementary Figures

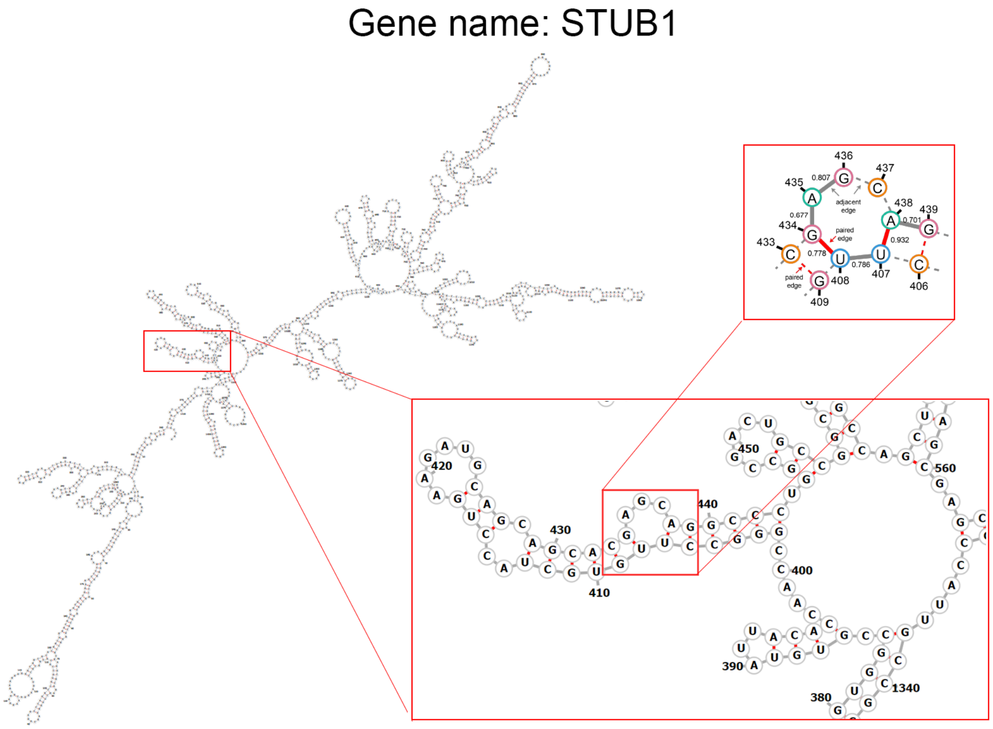

Fig. S1. Schematic representation of the secondary structure (predicted by RNAFold) of mRNA STUB1.

Gene name: HIST4H4

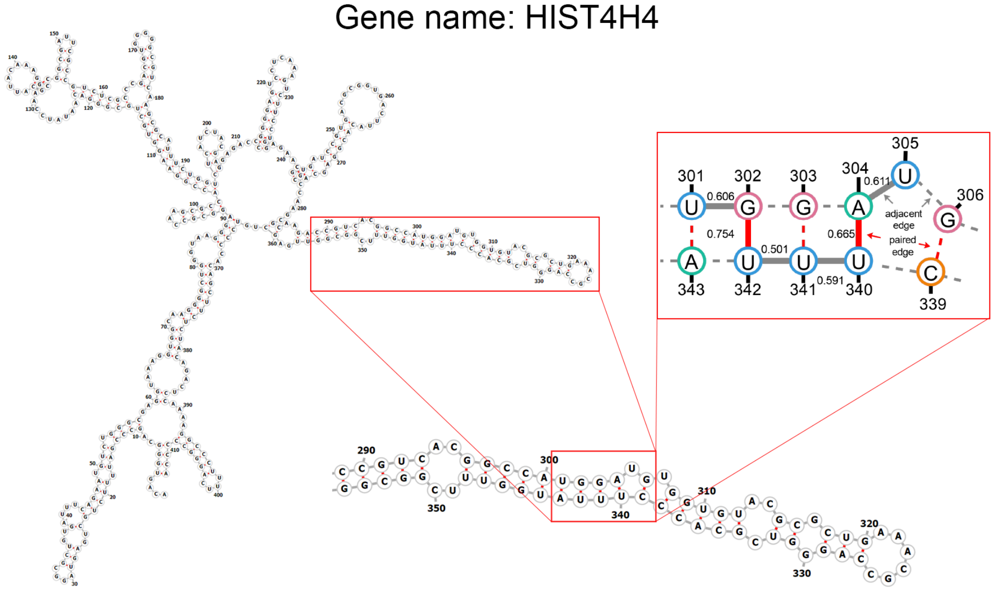

Fig. S2. Schematic representation of the secondary structure (predicted by RNAFold) of mRNA HIST4H4.

Gene name: TMEM238

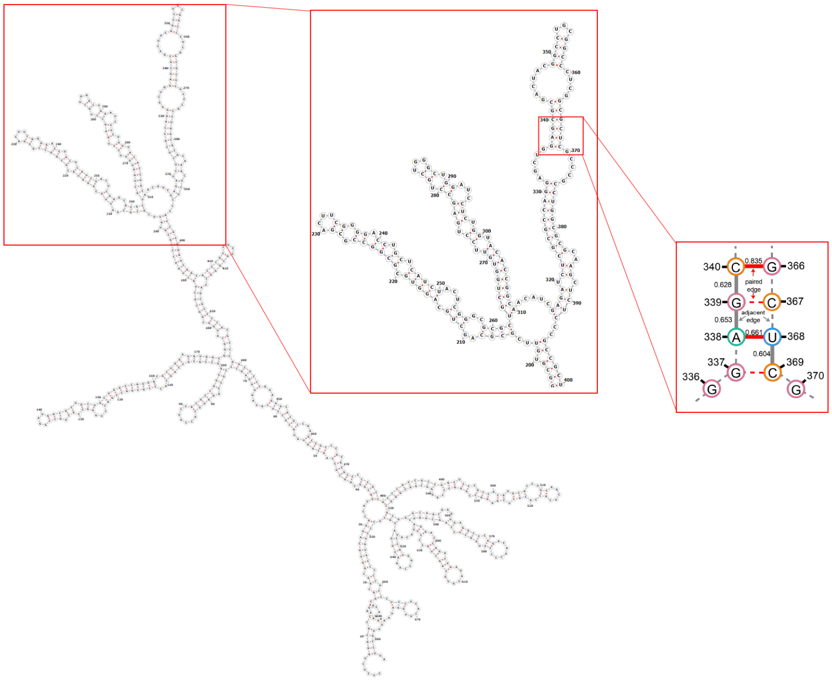

Fig. S3. Schematic representation of the secondary structure (predicted by RNAFold) of mRNA TMEM238.

Gene name: SPINK9

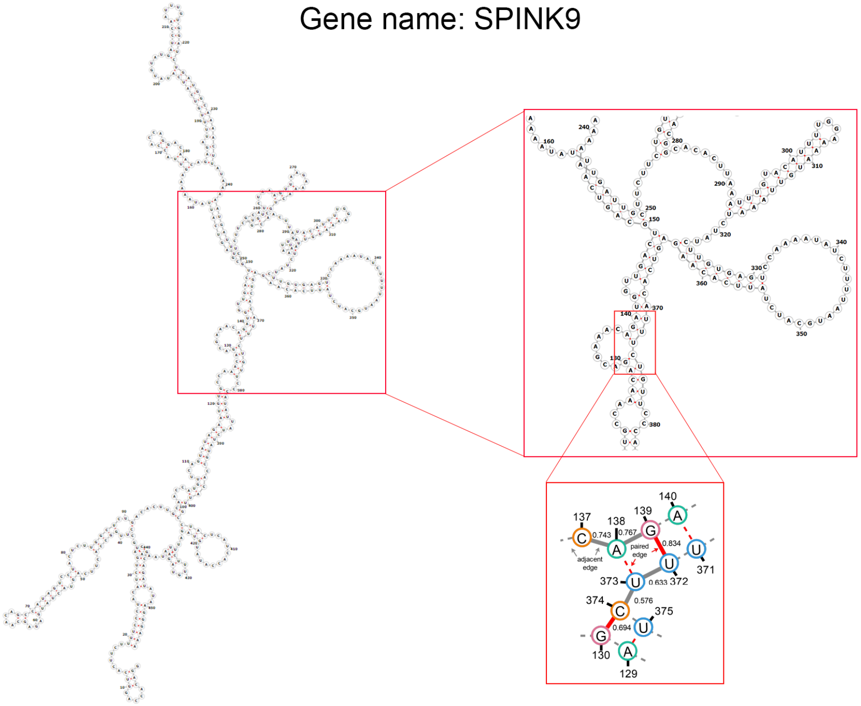

Fig. S4. Schematic representation of the secondary structure (predicted by RNAFold) of mRNA SPINK9.
